# Bone loss markers in the earliest Pacific Islanders

**DOI:** 10.1038/s41598-021-83264-3

**Published:** 2021-02-17

**Authors:** Justyna J. Miszkiewicz, Frédérique Valentin, Christina Vrahnas, Natalie A. Sims, Jitraporn Vongsvivut, Mark J. Tobin, Geoffrey Clark

**Affiliations:** 1 School of Archaeology and Anthropology, Australian National University, 44 Linnaeus Way, Canberra, ACT 2601 Australia; 2 CNRS, UMR 7041, ArScAn, Ethnologie préhistorique, Maison René-Ginouvès, Archéologie et Ethnologie, 21 Allée de l’Université, 92023 Nanterre Cedex, France; 3 Archaeology and Natural History, School of Culture History and Language, College of Asia and the Pacific, Australian National University, Canberra, ACT 2601 Australia; 4 Bone Biology and Disease Unit, St. Vincent’s Institute of Medical Research, 9 Princes Street, Fitzroy, Melbourne, VIC 3065 Australia; 5 Department of Medicine, St. Vincent’s Hospital, The University of Melbourne, Melbourne, VIC 3065 Australia; 6 MRC Protein Phosphorylation and Ubiquitylation Unit, James Black Centre, University of Dundee, Dundee, DD1 5EH UK; 7 Infrared Microspectroscopy Beamline, ANSTO - Australian Synchrotron, 800 Blackburn Road, Clayton, VIC 3168 Australia

**Keywords:** Bone, Obesity, Biological anthropology, Archaeology

## Abstract

Kingdom of Tonga in Polynesia is one of the most obese nations where metabolic conditions, sedentary lifestyles, and poor quality diet are widespread. These factors can lead to poor musculoskeletal health. However, whether metabolic abnormalities such as osteoporosis occurred in archaeological populations of Tonga is unknown. We employed a microscopic investigation of femur samples to establish whether bone loss afflicted humans in this Pacific region approximately 3000 years ago. Histology, laser confocal microscopy, and synchrotron Fourier-transform infrared microspectroscopy were used to measure bone vascular canal densities, bone porosity, and carbonate and phosphate content of bone composition in eight samples extracted from adult Talasiu males and females dated to 2650 BP. Compared to males, samples from females had fewer vascular canals, lower carbonate and phosphate content, and higher bone porosity. Although both sexes showed evidence of trabecularised cortical bone, it was more widespread in females (35.5%) than males (15.8%). Our data suggest experiences of advanced bone resorption, possibly as a result of osteoporosis. This provides first evidence for microscopic bone loss in a sample of archaeological humans from a Pacific population widely afflicted by metabolic conditions today.

## Introduction

The global obesity pandemic is increasing at an alarming rate posing growing health concerns and an economic burden for human generations today and in the future^1^. In 2019, the cost of treating obesity was estimated at USD $2 trillion (~ 2.8% of gross domestic product globally), with 9% of world population considered obese, and a further 392 million of youth predicted to become overweight or obese by 2025^1,2^. Human populations in the Pacific Islands are consistently classified as some of the world’s most obese nations^3^. The Kingdom of Tonga in Polynesia, in particular, has one of the largest prevalence in type 2 diabetes^4^. The reasons behind such high prevalence of metabolic conditions across the Pacific are complex. Studies have identified hereditary factors^3,5^ and environmental constraints that interplay with genetics, individual lifestyle, and access to resources^6,7^. While modern sedentary lifestyle is understood to underlie many metabolic conditions^8^, shedding light on the roots of these conditions can be predominantly achieved by examining health and disease markers left on human remains in the archaeological record^9,10^. Prior research examining the surviving skeletons of archaeological Pacific Islanders reported possible evidence of diseases such as gout^11^ and Forestier’s disease (diffuse idiopathic skeletal hyperostosis—DISH)^12^ in ca. 3,000‐year‐old Vanuatu. Both diseases occur in the same populations today^13^, which suggests that the origins of these cannot be completely modern. Therefore, the study of archaeological human skeletal remains has the potential to fundamentally shift our understanding of the origins, or at least continuity in occurrence, of modern health problems^14,15^^.^

Obesity is typically associated with physical inactivity and nutrition of poor quality^16^. Both factors can have detrimental effects on bone physiology and structure because of a minimised mechanical loading regime^17^ and restricted dietary calcium and vitamin consumption^18,19^, which can lead to an increase in bone turnover where bone loss dominates bone gain^18^. This advances bone tissue age and can result in greater fragility, susceptibility to fractures, and a diagnosis of osteopenia or osteoporosis^19^. The deteriorating bone microarchitecture is caused by a shift in the activity balance between cells that normally renew the skeleton by bone remodelling such that osteoclast-mediated bone resorption exceeds bone deposition by osteoblasts^20,21^. Continuing bone loss in such fragile bones of individuals, particularly the elderly, with untreated osteoporosis impairs lifestyle and mobility as fractures occur repeatedly or heal poorly. Osteoporosis is common in modern aging populations, and as such poses a large economic burden. The condition is known as the “silent disease” manifesting only once fracture has occurred^22–24^. The difficulty in completely preventing osteoporosis lies in its multi-factorial and inter-linked aetiology. Osteoporosis, meaning ‘porous bones’, can take different forms (e.g. primary or secondary) and as such develops as a result of various factors including ageing^25^, female biological sex^26^, lack of physical activity^27^, genetic predisposition^28^, hormone deficiencies^29^, dietary restrictions^18,19^, including those resulting from psychiatric conditions such as anorexia nervosa^30^, and a combination of these and other variables that can relate to socio-economic status^14,15^. The effect of sex on bone health has been particularly well studied in post-menopausal women, where loss of oestrogen, a hormone essential in inhibiting bone resorption, is a causative factor^31,32^.

There has been much discussion about the relationship between obesity and osteoporosis^33–36^. Not only does a lack of exercise negatively impact bone density, a cell based link between fat and bone tissue has been proposed^37^. As bone loss increases with age, the number of bone marrow fat cells (adipocytes) rises^37^. Bone-forming osteoblasts and fat cells differentiate from the same bone-marrow precursor cells, yet whether bone loss is a precursor to fat infiltration or vice versa remains a point of contention^37^. To complicate matters further, a heavier frame can have a positive effect on bone formation due to the higher weight-bearing from increased body mass—a relationship which has been termed the “obesity paradox”^35,36^. The development of osteoporotic bone in obese patients has been demonstrated in clinical studies, though data have not been consistent^38–40^. For example, some sites of the skeleton, such as vertebrae, but not the hip, have been classified as osteoporotic in obese or overweight individuals^39^^.^ Obesity incidence has been reported in Tonga^4^, but osteoporosis data are extremely limited. Reports from the past two decades paint a conflicting picture. In 2006, Tonga (included amongst other countries from the “Western Pacific”) was ranked second highest, after Europe, for prevalence of osteoporotic fractures^41^. In contrast, “the Polynesian bone phenotype” has been characterised as having higher bone density than that of European counterparts^42^. The International Osteoporosis Foundation is yet to map fracture incidence across the Pacific Islands comprehensively^43^. The efficiency of data collection there is possibly hindered by limited radiography infrastructure^42^.

Clinically, osteoporosis in modern populations is diagnosed from measures of bone mineral density (BMD) and occurrence of fractures^44–46^. Whether an individual suffers from abnormal bone fragility defined as osteopenia or osteoporosis is diagnosed from BMD T-scores that are lower than those of healthy age-matched cohorts^47^. A gold standard methodology to collect BMD in living people is dual energy x-ray absorptiometry (bone densitometry, DXA)^48^. Diagnosis can be supplemented through bone turnover markers from bone histomorphometry^49^, or quantitative ultrasound techniques^50^, and various other types of microscopy that measure bone porosity two- (2D) or three- (3D) dimensionally^51^.

Similar means have been implemented to confirm the incidence of possible osteoporosis in various archaeological European^52–57^, North American^58^, and African^59–61^ osteological collections (see reviews^15,62–64^). Where access to large and well-preserved specimens has been possible, DXA, cortical and/or trabecular bone volume, and bone turnover assessment methods have indicated significant bone loss^52–55,60,65–67^. Analyses of fracture types and their patterns (such as classic Colles’ fractures) have also provided insights into archaeological experiences of osteoporosis^66–69^. Microscopic methods reconstructing bone turnover, mineral composition, and trabecular and cortical bone microarchitecture in archaeological human remains have also been successful^70–72^. Non-invasive approaches such as 3D micro-computed tomography (micro-CT) can help in the calculation of trabecular or cortical bone thickness, separation, and the visualisation of porosity network^73,74^. Invasive methods such as histology rely on an extraction of samples to describe more localised bone changes^72,75^. Therefore, invasive techniques are less often applied to archaeological samples because of the irreplaceable nature of excavated remains^76^, and so typically rely on smaller sample sizes^77^. Additionally, preservation issues (such as degradation of bone microstructure post-mortem^78^) can further limit the application of modern clinical osteoporosis diagnostic criteria to archaeological remains^71,79^. Therefore, while the examination of disease in archaeological remains can provide fruitful results, response of living bone to disease cannot be observed. However, a combination of different methods can provide multiple lines of data for differential diagnosis and interpretation.

**Figure 1:**
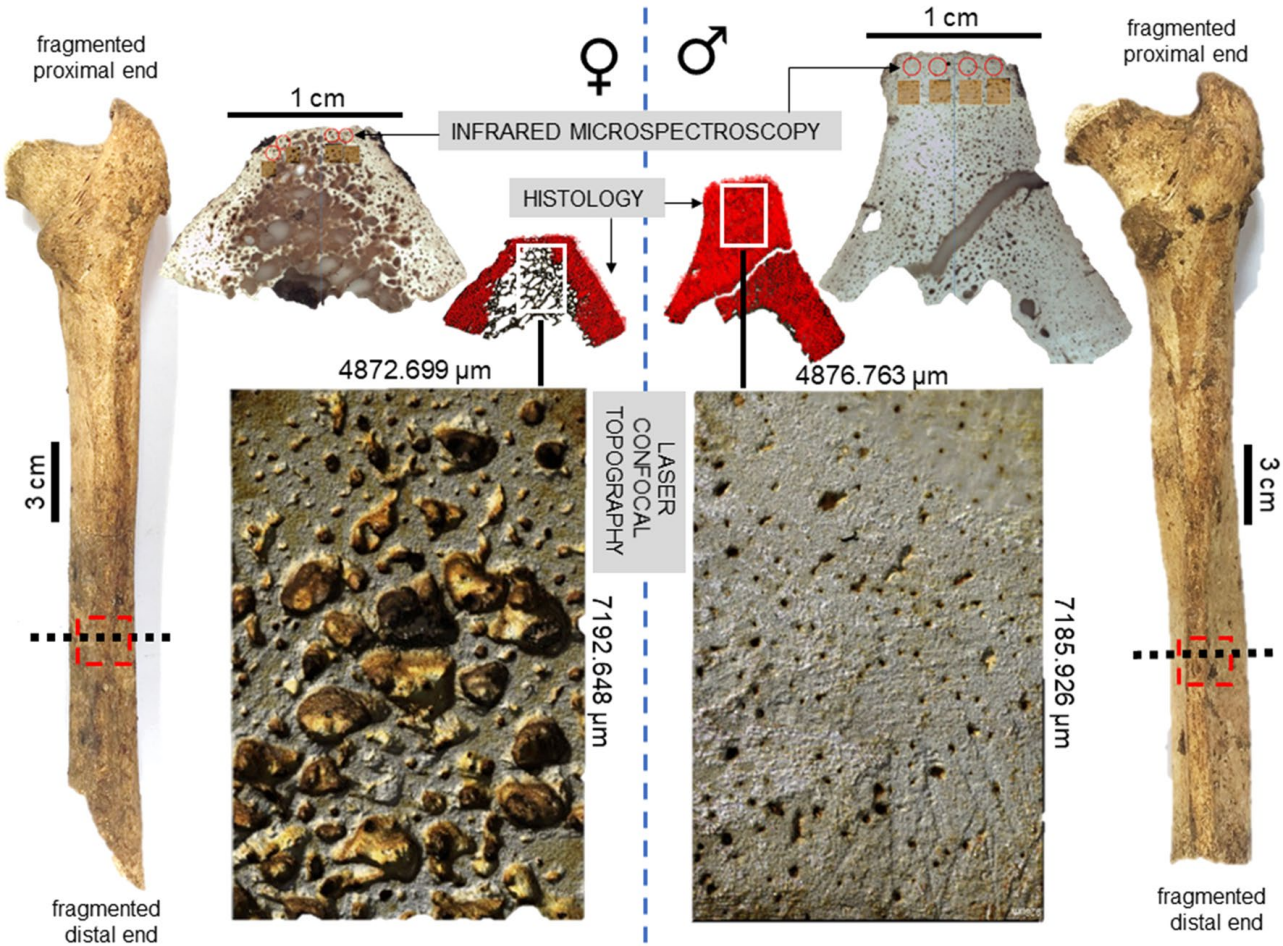
Summary of methods and key findings in the present study. Posterior view of two right archaeological femora from Talasiu individuals estimated as female (♀, ID: Sk3.1) and male (♂, ID: Sk3.2) shows the sectioning location (black dashed line) and approximate sample removed (red dashed box). Methodological steps included synchrotron sourced infrared microspectroscopy to measure bone mineral composition, histomorphometry to estimate Haversian canal densities (red dots), and laser confocal scanning of bone topography to provide a qualitative illustration of intra-cortical bone porosity producing trabecularisation.

**Figure 2:**
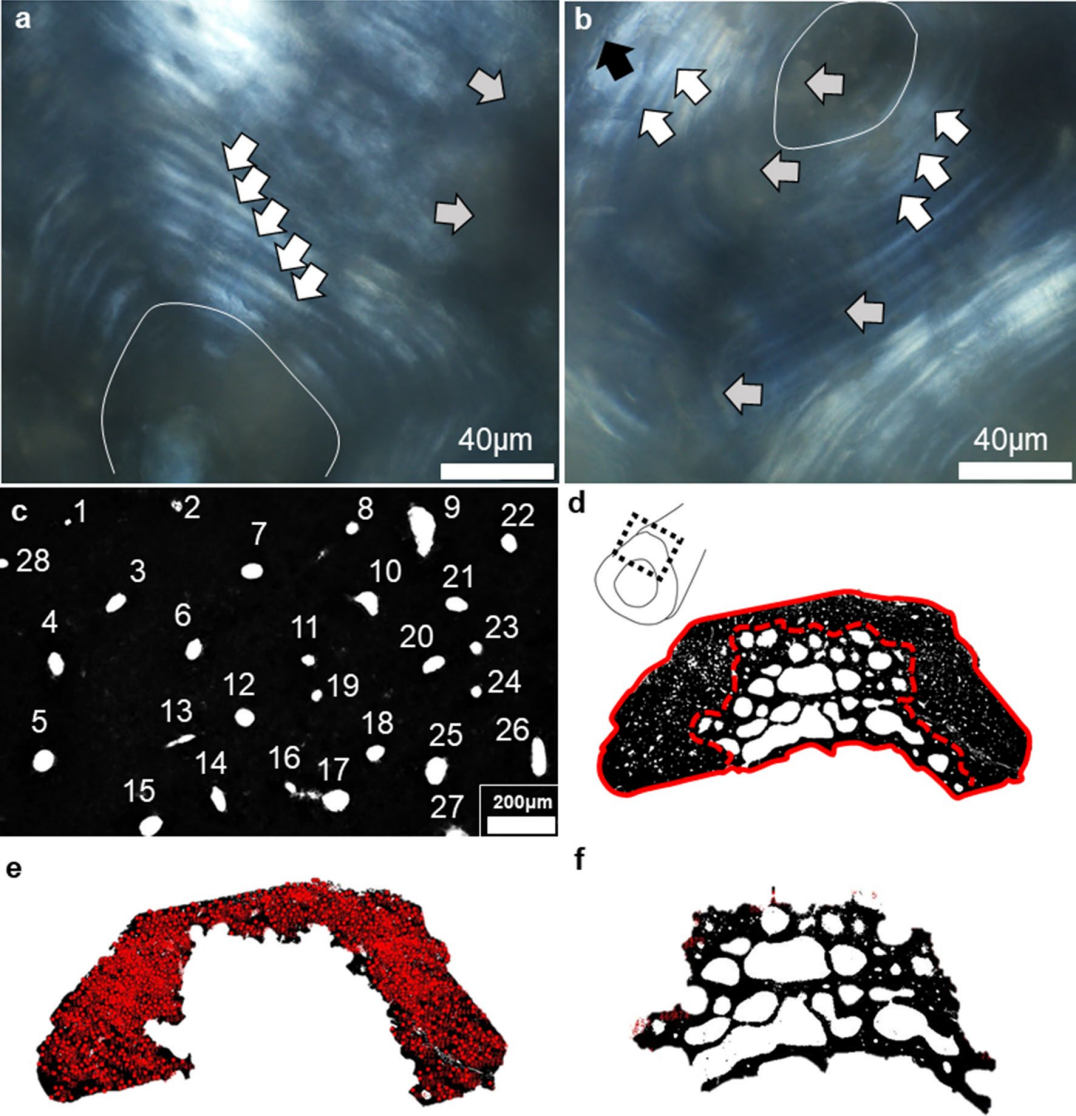
A summary of histomorphometric procedures for estimating densities of Haversian canals and abnormal intra-cortical bone porosity. (**a**, **b**): localised histology (60 ×) viewed under linearly polarised light showing secondary osteon lamellae (white arrows) surrounding Haversian canals (oval features), which were obscured by patches of diagenesis (grey arrows). Cement lines (black arrow in **b**) were inconsistently preserved (examples shown are from individuals BG2 (**a**) and Sk9.3 (**b**). Haversian canals were counted manually form thresholded images (**c**–**f**): (**c**) shows numbered canals, (**d**) shows manual segmentation of cortical wall from abnormal intra-cortical porosity (red dashed line), while the solid line marks whole section area in individual BG2; (**e**) illustrates final counts of Haversian canals, while (**f**) shows ‘extracted’ abnormal bone porosity.

**Figure 3:**
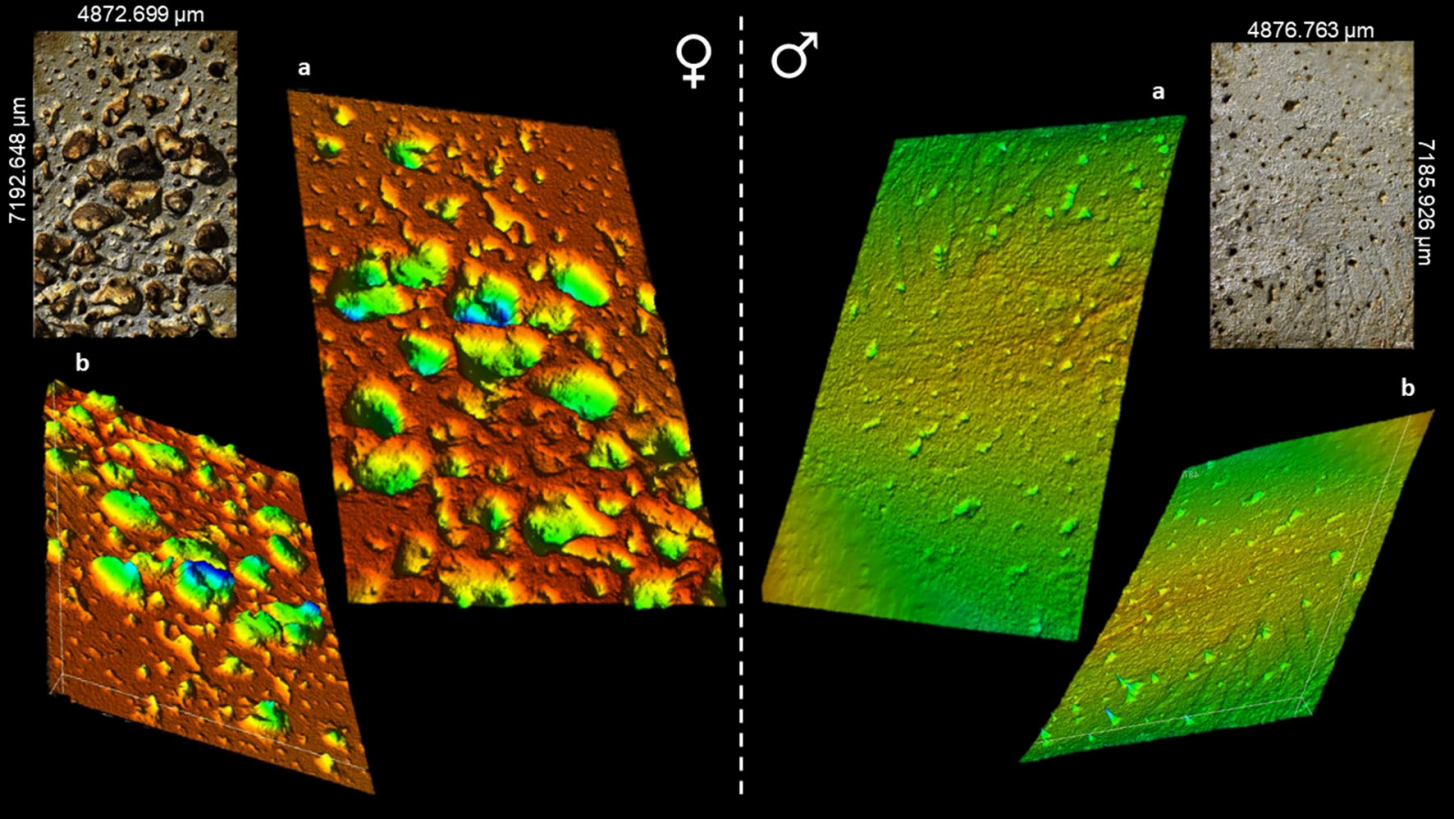
Intra-cortical surfaces scanned using a three-dimensional laser confocal topography microscope Olympus OLS5000 with applied false-coloured heat map to illustrate extreme differences in porosity in archaeological posterior midshaft femur samples from Talasiu individuals estimated as female (♀, ID: Sk3.1) and male (♂, ID: Sk3.2). (**a**) shows topography viewed superiorly from the bone surface, whereas (**b**) is reverted upside down to show the inferior view of the same scan as (**a**).

To the best of our knowledge, bone loss as a result of possible osteoporosis has not yet been investigated in archaeological samples in the Pacific. Obesity specifically as a factor leading to osteoporosis cannot be directly reconstructed for archaeological specimens as the available methods of body mass estimation still provide large error^80^. However, given the widespread health issues in contemporary Pacific nations, bone loss markers in archaeological human remains in this region could be an indicator of musculoskeletal health and metabolic issues similar to those seen there today. The 2,650 BP archaeological site of Talasiu in the Kingdom of Tonga is one of the most significant sites ever uncovered in the Pacific^81,82^. This site has previously produced the first evidence for Early Polynesian mortuary behaviour^81,82^, archaeological Polynesian diet relying on marine resources^83^, and insights into the abandonment of ceramics by early Polynesian people^84^. We set out to test whether archaeological Tongan human bone would show evidence of abnormal bone loss marked by sex-specific differences. We employed an invasive microscopic examination to characterise bone metabolic fluctuations in eight samples extracted from femora in the Talasiu individuals, sub-divided into groups of estimated sex (four males, four females). All individuals were classified as ‘adults’ who likely lived beyond 30 years of age, which means their bone tissue had entered an age-driven degeneration phase following a peak bone mass accrual point of lifespan. One female (ID: Sk3.1) was possibly an elderly individual (Table S1), who might have survived into a sixth life decade and possibly beyond. We used several microscopic methods to provide multiple lines of data. We recorded sexually dimorphic femur size differences, and then combined 2D histology^75,85^, 3D laser scanning confocal microscopy^51^, and synchrotron particle accelerator methods (Fourier-transform infrared microspectroscopy, sFTIRM)^86,87^ (Fig. 1). This allowed us to examine compact femur bone Haversian canal densities as a proxy for remodelling (Fig. 2), abnormal porosity as a marker of prolonged bone resorption (Figs. 2, 3), and bone matrix composition through carbonate and phosphate content (Fig. 1), averages of which we hypothesised to differ between the estimated female and male sex groups.

## Results

We found evidence for bone loss, which differed between the estimated sex groups in our samples. Porosity which manifested as cortical bone trabecularisation was particularly advanced in the females. As expected, due to sexual dimorphism, males in our sample had larger femora when compared to the females (Table 1, Fig. 1). The average midshaft circumference was 92.25 mm (standard deviation (SD) = 4.79) in males, but 79.75 mm (SD = 7.09) in females (*U* = 15.5; *p* = 0.029). The diameter of femur midshaft was also larger and more pronounced in males in both the antero-posterior (A-P), and medio-lateral (M-L) planes. The A-P diameter was 30.75 mm (SD = 2.87) in males, but 27.26 mm (SD = 2.15) in females, albeit this difference was not statistically significant.

**Table 1:** Sample details and raw gross morphometric data of the femora representing adult archaeological males and females from Talasiu, Tongatapu, Kingdom of Tonga. Cortical width and midshaft circumference are reported in mm. A-P: antero-posterior, M-L: medio-lateral, H.Dn: Haversian canal density per mm^2^, %Po.Ar: percent of sample impacted by porosity producing trabecularisation.

Individual ID	Estimated sex	Cortical width		Midshaft circumference	Quantitative histology	
		A–P	M–L		H.Dn	%Po.Ar
BG2	Female	25.28	23.35	76	22.223	31.890
Sk3.1	Female	28.93	24.87	86	13.159	50.232
Sk3.2	Male	33.87	29.27	101	15.627	7.261
Sk5	Male	27.72	22.17	86	20.798	0.000
Sk9.2*	Female**	29.30	26.54	81	14.815	46.001
Sk9.3	Male**	29.00	27.34	87	16.530	51.793
Sk12	Female**	25.54	23.26	76	12.216	13.908
Sk14	Male	32.41	26.92	95	16.826	4.197

*left femur, **probable sex.

**Figure 4:**
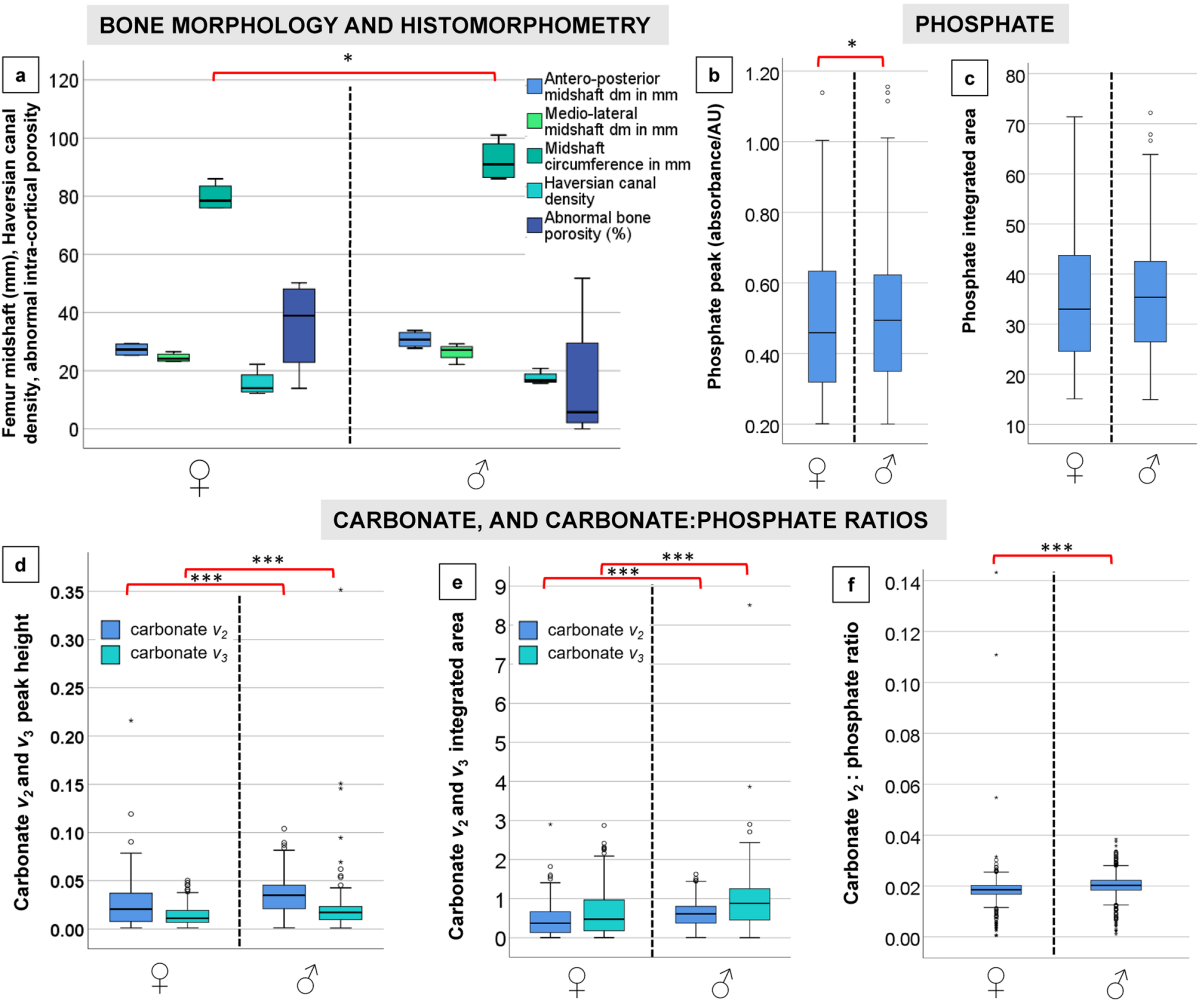
Graphical summary of results illustrating descriptive comparisons of data between archaeological Tongan individuals of estimated male (♂) and female (♀) sex. The boxplots indicate that females present with: (**a**) smaller femoral midshaft (dm: diameter), with lower Haversian canal density (per mm^2^), but higher intra-cortical porosity producing trabecularisation (%); (**b**, **c**) lower phosphate content; and (**d**–**f**) lower carbonate, and carbonate:phosphate ratios. Outliers are marked by asterisks and circles. *p < 0.05; ***p < 0.0001.

**Figure 5:**
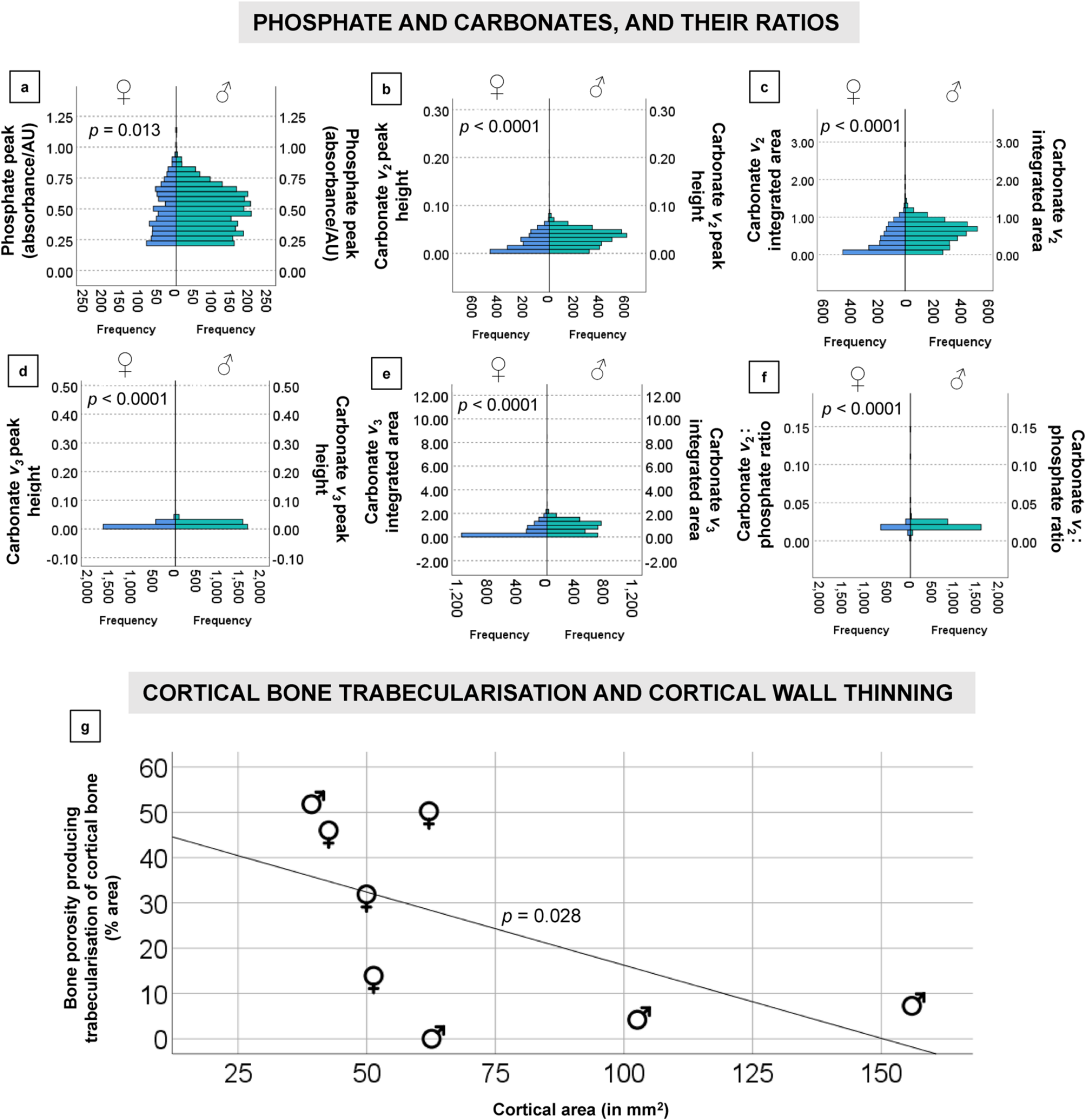
Statistically significant Mann Whitney *U* ranking comparisons of the sFTIRM data for phosphate peaks (**a**), carbonate peaks and integrated area (**b**–**e**), and ratios computed using carbonate *v*_2_: phosphate area (**f**). A strong negative correlation between cortical area and trabecularised cortical bone in the whole sample is shown (**g**).

The sexual dimorphism was likely also reflected in the 2D histological measurements of bone Haversian canal density collected from the cortical area of bone unaffected by porosity producing trabecularisation, which was higher in males (17.445/mm^2^) than in females (15.603/mm^2^) (Table 1, S2; Figs. 1, 2, 4). However, the area of bone samples showing porosity producing trabecularisation which represented as ‘giant’ coalescing pores was more evident in females than in males (Fig. 1). The average porosity producing trabecularisation was 35.5% (SD = 16.40) in females, but only 15.8% (SD = 24.17) in males. In fact, all four female samples were affected by a high degree of porosity producing trabecularisation that was widespread from the most inner bone layer (endo-cortical) through the mid-section region (intra-cortical), reaching the bone areas immediate to the most outer bone layer (periosteum) (Figs. 1, 2). This led to cortical wall thinning^88, 89^ and was a consistent pattern throughout the sample as supported by a statistically significant negative correlation between percent areas of trabecularisation and cortical wall area (*Rho* = − 0.762, *p* = 0.028; Fig. 5). However, comparing only the porosity and Haversian canal variables between the two sex groups did not result in statistical significance as males were clearly impacted by intra-cortical trabecularisation as well. In fact, one of the male samples (ID: Sk9.3, Figure S1) showed an estimated 51.8% of trabecularisation of intra-cortical bone. This data point is maximum across our entire sample of eight bones. The remaining three male samples showed an overall consistent appearance of porosity whereby the bone surfaces were dense, showing a limited range of variably sized porosity regions and their occurrence (one complete absence, Figs. 1, 3). On the contrary, the female samples exhibited much more variability in porosity producing trabecularisation (Table 1, S2; Figs. 1, 2), ranging from 13.9% minimum to 50.2% maximum of cortical bone space. The 3D confocal laser scans of bone surfaces from two cases showing extremes in bone micro-organisation (one female Sk3.1, one male Sk3.2) (Figs. 1, 3, S10, S11) demonstrated an almost entirely limited topography in the male sample, but more variable topography in the female sample which was punctuated with relatively deep and ‘giant pores’ the bone cortex.

The bone carbonate and phosphate content measured through sFTIRM was consistently lower in females (Figs. 4, 5; Table 2, S3–S6) (*p* < 0.05 in all sFTIRM variables except for integrated phosphate area). The average peak height of phosphate (from 848 spectra; *U* = 1,118,955.000; *p* < 0.013), and *v*_2_ (from 1676 spectra; *U* = 3,767,893.000; *p* < 0.0001) and *v*_3_ carbonates (from 2159 spectra; *U* = 4,978,269.000; *p* < 0.0001) were lower in females. While the phosphate difference of 2.6% in the means seems marginal, it was more substantial when considering *v*_2_ (30.3%) and *v*_3_ (32%) carbonates. We note that more spectra were successfully measured in the samples from males (2496 phosphate spectra, minimum 3329 *v*_2_ and 3264 *v*_3_ spectra) (Table 2, S3–S5). Compared to the male samples, the average integrated areas of phosphate and two carbonate peaks were also lower in females. The phosphate area in females was only lower by 1.1% (Table S3). However, the *v*_2_ (*U* = 3,765,291.500; *p* < 0.0001); and *v*_3_ (*U* = 4,847,614.000; *p* < 0.0001) carbonates were 35.3% and 44.12% greater in the samples from males (Table S4, S5). The carbonate *v*_2_:phosphate integrated area ratios from the female samples were lower by 5% when compared to males (*U* = 1,421,109.000; *p* < 0.0001; Table S6). This was despite females recording a maximum ratio spectrum of 0.143 contrasted with a much lower maximum spectrum of 0.038 in males, and the higher total number of spectra collected in males (2495) contrasted with 846 spectra in females.

**Table 2:** Histomorphometry and synchrotron source Fourier-transform infrared microspectroscopy descriptive data per estimated sex (four males and four females). SD: standard deviation, min.: minimum data, max.: maximum data, A-P: antero–posterior, M-L: medio-lateral, H.Dn: Haversian canal density per mm^2^, %Po.Ar: percent area of sample impacted by porosity producing trabecularisation.

Estimated sex	N	Min	Max	Mean	SD
**Femur size and quantitative histology**					
**Female**					
A-P midshaft diameter (mm)	4	25.28	29.30	27.26	2.15
M-L midshaft diameter (mm)	4	23.26	26.54	24.51	1.54
Midshaft circumference (mm)	4	76.00	86.00	79.75	4.79
H.Dn (/mm^2^)	4	12.216	22.223	15.60	4.54
%Po.Ar	4	13.908	50.232	35.508	16.397
**Male**					
A-P midshaft diameter (mm)	4	27.72	33.87	30.75	2.87
M-L midshaft diameter (mm)	4	22.17	29.27	26.42	3.02
Midshaft circumference (mm)	4	86.00	101.00	92.25	7.09
H.Dn (/mm^2^)	4	15.63	20.80	17.45	2.295
%Po.Ar	4	0.000	51.793	15.813	24.171
**Fourier-transform infrared microspectroscopy**					
**Female**					
Peak phosphate height	848	0.201	1.139	0.481	0.189
Phosphate integrated area	848	15.087	71.381	34.518	11.673
Carbonate *v*_2_ height	1676	0.001	0.216	0.023	0.018
Carbonate *v*_2_ integrated area	2159	0.001	2.899	0.392	0.327
Carbonate *v*_3_ height	1676	0.001	0.050	0.011	0.008
Carbonate *v*_3_ integrated area	2071	0.001	2.873	0.475	0.498
Carbonate *v*_*2*:_ phosphate ratio	846	0.001	0.143	0.019	0.007
**Male**					
Peak phosphate height	2496	0.200	1.156	0.494	0.171
Phosphate integrated area	2496	14.937	72.184	34.899	10.346
Carbonate *v*_2_ height	3339	0.001	0.104	0.033	0.017
Carbonate *v*_2_ integrated area	3339	0.003	1.625	0.576	0.303
Carbonate *v*_3_ height	3329	0.001	0.352	0.017	0.011
Carbonate *v*_3_ integrated area	3264	0.001	8.510	0.850	0.526
Carbonate *v*_*2*:_ phosphate ratio	2495	0.001	0.038	0.020	0.003

## Discussion

We report the first microscopic record of bone loss characteristics in archaeological femora representing some of the earliest Pacific Islanders. On the basis of how commonplace contemporary metabolic issues, including obesity and diabetes, are in Tonga, we interpret our evidence to indicate that similar problems that might have led to osteoporosis occurred there ca. 3000 years ago. Two key implications are presented. Firstly, our small sample of archaeological humans from the Pacific appears to be afflicted by cortical bone porosity producing trabecularisation despite the notion that osteoporosis and related conditions are modern-day diseases. This confirms that the occurrence of widespread metabolic disease in this region today may have roots in the past. It agrees with bone loss experiences reported for archaeological collections from around the world (see reviews^15,62–64^), and archaeological metabolic bone disease indicators reported in other parts of the Pacific^11,12^. Secondly, the extensive cortical bone porosity leading to trabecularisation in our sample of archaeological Tongan females suggests experiences of oestrogen loss, mirroring modern day female osteoporosis incidence. This may further suggest at least the females represented in our sample lived to menopause age. The occurrence of trabecularisation in some of the male samples further points to advanced age despite widely held assumptions that past human longevity was shorter than today. We are unable to ascertain that archaeological Tongans specifically suffered from obesity, but we explain our findings through a differential diagnosis considering various aetiologies that might have impacted these individuals—complementarily or in an alternate fashion.

### Occurrence of metabolic abnormalities as a result of lifestyle and environmental factors

The examination of human remains from archaeological backgrounds, such as tracing the origins of tuberculosis or leprosy^90^, has revolutionised our current understanding of disease in the modern world. Osteoporosis is often considered a modern disease because it mostly manifests in the elderly and is strongly linked to modern sedentary lifestyles. However, its incidence was previously confirmed in various archaeological European^52–57^, North American^58^, and African^59–61^ osteological assemblages. We now report the first microscopic record of abnormally porous human bones from the Pacific region, adding to the growing body of evidence that modern metabolic conditions in the Pacific may be rooted in the past^11,12^.

Talasiu people are some of the earliest representatives of the Polynesian society emerging after the first settlement of archipelago at c. 2850 cal BP^84^. The estimated Tongan skeletal fragility presented here is consistent with a historic basis for other current highly prevalent widespread metabolic problems such as obesity and type 2 diabetes in the region^3–5^. Published evidence also exists for other types of skeletal abnormalities reported from different archaeological sites in Tonga^91–94^, and Vanuatu^11,12,95^. For example, scurvy and hypervitaminosis A, and iron-deficiency anaemia were discussed amongst several possible conditions arising in archaeological Tongan communities possibly as a result of poor nutrition^91^. This came from skeletal evidence in 17 children aged 6 months to 3 years old suggesting a combination of infections and metabolic bone disease at the ’Atele pre-European burial mounds dated to AD 1100–1250 from Western Tonga^91^. Further, poorly mineralised 21 archaeological deciduous teeth obtained from infant skeletons excavated at ‘Atele in Tonga also indicated developmental disturbances in dental enamel linked to physiological stress arising from possible infections and nutritional deficiencies^92^. Stable isotope data extracted from archaeological human Tongan bone suggested diets to be predominantly based on starchy and marine food sources^83,93^. Selection of a wider variety of food sources would have been limited by the island size in addition to norms arising from social organisation^83^. Another site in Tonga, ca. 750–150 BP To-At-36 at Ha ‘ateiho, yielded examples of dental developmental disturbance and growth disruption indicators in 33 adult and 11 juveniles^94^. Lower spine degeneration (spondylosis) was also observed on archaeological Tongan lumbar vertebrae^94^. While spondylosis occurring as a result of bone degeneration due to age can be accelerated through weight bearing from active lifestyles^94^ (combat sports at archaeological Tonga mounds were previously also discussed^96^, and see our next discussion section about physical activity links to age-related bone loss), clinical studies have also linked it to increased body weight in obesity^97^. The experiences of degenerative bone conditions in our sample of the Tongan individuals can be inferred from joint lesions indicating osteoarthritis (Table S1).

In Vanuatu, an archipelago western to Tonga, previous reports of most likely diagnoses of scurvy due to vitamin C deficiencies^95^, gouty arthritis and DISH^11,12^, point to significant metabolic and nutritional issues afflicting Pacific Islanders thousands of years ago. While we cannot provide one aetiological explanation for the occurrence of bone metabolic conditions in our sample, we propose that a combination of lifestyle factors such as nutrition, and genetic predisposition played a role in the poor Tongan bone metabolism. Theoretical attempts to explain the high incidence of metabolic disease in the Pacific have predominantly focused on genetics (see^98,99^ for discussion). “The Thrifty Genotype” hypothesis proposes that thrifty genes, which would have been advantageous during famine events in the human past, drive diabetes and obesity in modern environments^99^. However, considering our data, and the aforementioned archaeological skeletal data from other sites in the region^11,12,91–95^, it is clear that genes alone cannot elucidate the human obesity trends in the Pacific. Additionally, as noted previously by Gosling et al^98^, thrifty genes cannot alone account for a range of selective pressures that characterised the Pacific islands. Alternative, or complementary, interpretations to consider include early childhood exposure to other diseases^91,92,100^, compromising adult immunity which can equally result in altered bone turnover and poor skeletal mineralisation^100,101^. Ongoing macroscopic pathology examination of the Talasiu sample will provide further data and help shed light on possible incidence of conditions such as DISH or gout, expanding our reported microscopic bone loss markers.

### Sex-specific and ageing driven bone fragility

Osteoporosis has a long history of afflicting females more than males due to menopause-driven loss of estrogen^31,32^. Oestrogen plays a key role in inhibiting osteoclast-mediated bone resorption, by regulating osteoclast apoptosis^31^. After menopause, prolonged bone loss occurs leading to increasingly porous and weak bones. Females are biologically disadvantaged because of this phenomenon, with modern postmenopausal women experiencing four times the level of osteoporosis than men^102^. Our data for a sample of archaeological Tongan females appear to match this sex-specific difference in bone fragility, providing another line of interpretation as an alternative or complementary aetiology. All our female samples showed a trabecularisation effect whereby the compact bone exhibited trabeculae-like architecture accompanied by cortical wall thinning (Figs. 1, 2). Female samples exhibiting higher cortical bone loss than male samples as a result of sex differences is consistent with prior archaeological^58^ and clinical reports^103–105^. The cortical wall bone not impacted by abnormal/trabecularised porosity in the female samples showed a lower density of Haversian canals, which can be interpreted as a proxy for the amount of remodelled bone^58,85^. This can be explained by males possibly experiencing higher mechanically stimulated bone remodelling than females, body size differences, and/or within-sample age differences as noted in prior studies^73,103,104,106^.

Inter-woven with the sex-specific differences in bone microstructure is ageing^65,72,103–105^. It is well understood that the variability of age impacts on bone structure with sex manifests substantially in the femoral cross-section (and in other lower limb bones)^107^. Ageing of bone tissue results in a greater resorption on the endocortical surface, leading to modified long bone cross-sectional geometric properties^107^. In a classic study examining this in a sample of US human cadavers^107^, age-related endocortical resorption manifested both in males and females. However, males appeared to exhibit a simultaneous formation of bone sub-periosteally and resorption endo-cortically, which did not drastically impact bone strength^107^. In females, while the medullary cavity expanded, there was no associated expansion of the sub-periosteal bone, meaning that their cross-sectional properties weakened with age^107^. When considering preindustrial humans such as the Pecos who undertook high levels of physical activity^108^, sex-differences in bone loss while present were not as extensive as those seen in the aforementioned cadavers^107^. In the Pecos, sub-periosteal bone and biomechanical properties of the femur (and tibia) followed a general pattern of increase with age in both sexes^108^. We were not able to undertake a biomechanical analysis of cross-sectional geometry, but the substantial thinning of cortical wall in the Tongan female samples, along with lower Haversian canal densities, could suggest not enough mechanically stimulated bone remodelling around the time of their peak bone mass accrual phase of the life-course (third life decade)^109^. Indeed, sedentary lifestyles are so widespread in Tonga today that several modern intervention efforts targeting adolescents have been unsuccessful^110^. This raises an important consideration for future studies of archaeological bone loss comparing populations from different parts of the world, whereby environmental and lifestyle factors impacting bone building in the early ontogeny will differ in accordance to genetic and cultural determinants^109^.

One of the negative repercussions of overall ‘improved’ longevity and mortality across contemporary human populations is the advanced age of soft and hard tissues that results in multiple degenerative diseases experienced by the elderly^111^. Bone fragility, increased fracture incidence, and hip-replacement surgeries, are some of the most common issues for the elderly of significantly deteriorated skeletal health. One of our female individuals (ID: Sk3.1) showed highly advanced skeletal deterioration characteristics (e.g. loss of all mandibular teeth ante-mortem, Figure S2) so she was classified as the oldest, and likely the only elderly, individual in our sample. This implies she might have survived into her sixth life decade (and possibly beyond). The combined age-at-death data and her abnormal intra-cortical porosity, are evidence that, at least some, archaeological Tongan females might have well surpassed the commonly assumed short longevity of humans in the distant past^112,113^. The trabecularisation of the cortical bone in our samples is remarkably similar to histology and microradiography images of midshafts examined in modern Australians aged 89 year old^88^, and 67, 78, 90 year old^114^. This encourages further research combining bone biology, skeletal anatomy, archaeology, and social structures of Tonga to elucidate aspects of care of elderly in past Polynesia^115^.

Archaeological context-specific and methodological limitations of our study mean we cannot provide a single diagnosis and aetiology of the bone loss characteristics reported. Access to hundreds of well-preserved skeletons, as has been the case in some prior archaeological osteoporosis studies^54,55^, is not possible at Talasiu as the site is on a remote island constrained by its land and population size. Given the archaeological age of the Talasiu samples, the preservation of bone is not comparable to modern or post-mortem tissue, and thus cannot be experimentally examined to the same level. Future microscopic research applied to archaeological human remains from the Pacific will hopefully generate more comparative data, ideally using complementary 3D and 2D methods where feasible. While our study is limited by the broad age-at-death ranges, this is an issue impossible to overcome in biological anthropology as a narrow and exact chronological age for archaeological individuals cannot be ascertained from gross osteological analysis alone^116^. The assignment of biological sex is also a probability estimate based on well-established sexually dimorphic features of the human skeleton, which, without future aDNA validation, will be the best sex estimate possible.

## Conclusions

Our results are the first microscopic record of cortical bone loss in a sample of archaeological humans from Tonga. Given the small sample size, we suggest caution in the generalisation of our results in regards to the wider archaeological Tongan societies. With larger archaeological samples from across the Pacific islands, a pattern in bone loss may be shown in the future. Nevertheless, our results are evidence that a possible occurrence of bone metabolic conditions in a sample of archaeological Pacific individuals can be detected microscopically. We think this is an important step forward for discussions about metabolic diseases in the past and present Pacific. We discussed several explanations for the observed bone loss markers including: sex-specific driven bone micro-architectural deterioration with the possibility of our sample of Tongan females experiencing hormonal changes due to menopause; age-related bone loss similar to that seen in modern aging populations; and lifestyle factors such as poor physical activity and nutrition. Equally, other diseases in the region compromising immunity might have contributed to abnormal bone physiology in our sample. Given how commonplace contemporary metabolic issues, including obesity and diabetes, are in Tonga, our key interpretation is that similar problems might have led to osteoporosis in our sample there ca. 3,000 years ago.

## Methods

We implemented an invasive methodology and so were restricted to eight femora (seven right and one left) (Table 1). The choice of femur side was determined on the basis of macroscopic preservation and availability of midshaft bone for extraction. Brief summarises of gross anatomically visible skeletal lesions per individual are reported in Table S1, with several individuals (6/8) showing evidence for osteoarthritis. While limited, this sample size is comparable to previous research implementing microscopic methods of bone assessment in archaeological samples^77,85^. The biological sex of the individuals represented by the femora had been previously estimated^82,83^ following established methods^117^. Four males and four females (including one probable male and two probable females, Table 1) were estimated. The inclusion of ‘probable’ sexes is standard practice where archaeological human remains that do not show a directional consistency in sexually dimorphic skeletal features^119^. The adult age-at-death was assigned based on morphological features of the pelvic auricular surface^118^. The true chronological age of these individuals cannot be ascertained from bone morphology alone.

The skeletal remains represented by the specimens reported here were excavated and analysed with the permission from the Lapaha Community and Nobles, and the Ministry of Internal Affairs (Government of Tonga). The samples are curated at the Australian National University, Canberra until further repatriation notice. Talasiu on Tongatapu in the Kingdom of Tonga is one of the most archaeologically significant sites in Asia–Pacific. It represents some of the earliest occupations of the Neolithic people migrating into the Pacific alongside the Lapita culture some 3200–2850 cal BP^120^. It is a shoreline site located to the north of Lapaha, and is mainly comprised of shell middens and ceramic deposits^115^. The first excavation of Talasiu had taken place in 1957 which was subsequently followed by dating of the recovered shellfish to 2800 + /− 70 BP^120^. Excavations from 2008 onwards revealed burials containing human remains, including burnt remains that provided an insight into early mortuary behaviours in the region^81^. At least 19 late-Lapita/ immediately post-Lapita single to multiple burials have been reported, detailing a possible total number of 45 individuals represented and complex mortuary practices^81,82^. Shell midden samples from Talasiu indicate a sedentary human occupation dated to approximately 2700–2650 cal BP^120^, with more recent calibrations of 2650 BP for the burials^82^. Twenty-one of these individuals were recently examined as part of a multi-isotopic analysis reconstructing the first Polynesian diet^83^. A sub-sample of these Talasiu human individuals with well-preserved femora was studied here to conduct the analysis of bone loss markers. Applying the microscopic methods reported here to bones previously sampled for isotopic analysis, and limiting sample extraction to a cortical quadrant (rather than a complete long bone cross-section) ensures minimal invasion of the archaeological human material as per ethical recommendations^76^.

Samples were collected from the midshaft femur due to its biomechanical versatility, and prior published data reporting abnormal porosity occurring there^103,104^. Prior to sampling, each femur was photographed and measured at midshaft using standard WorkZone calipers and a measure tape to obtain A-P and M-L diameters (in mm), and the midshaft circumference (in mm) (Table 1). As the femora were fragmented on the distal and proximal ends, we could not measure maximum femoral lengths (Fig. 1). However, the determination of the midshaft location was easily achieved by locating the *linea aspera*, which is also the region from which samples were extracted (Fig. 1). This followed prior methods examining femur bone histology in archaeological specimens^75,85^, and reports of increased porosity on the posterior aspect of the femur in modern cadavers^103^. Approximately 1 cm thick cortical quadrants were removed using a Dremel 200/2–30 Two-Speed Rotary Tool equipped with a rotary blade. Cutting was performed as per prior methodologies^121^. The samples detached loosely after the blade had reached the medullary cavity having made two parallel longitudinal and transverse cuts on the bone exterior.

The preparation for three different microscopic analyses (2D histology, 3D confocal laser topography scanning, sFTIRM, Fig. 1) included sequential partitioning of bone samples into slices. Using the Dremel blade, each sample was further cut in half to designate an approximately 0.5 cm thick bone section for confocal topography scanning. The remainder of the sample was embedded in Buehler epoxy resin to impregnate the internal bone structure for histology and sFTIRM. Using a Kemet MICRACUT precision cutter equipped with a 150 mm diamond blade, a ~ 150 μm thin slice of bone was removed from the embedded block and designated for sFTIRM. The remaining portion of the sample was set aside for histological preparation.

The preparation of thin sections followed standard methods applicable to archaeological human bone^75^. The histology surface revealed by cutting on the low speed cutter was glued to a microscope glass slide using Stuk epoxy glue. It was further trimmed on the saw and ground using a series of silicon carbide pads until ~ 100 μm thickness was reached. The sections were then polished using Buehler MicroPolish alumina powder and cleared in an ultrasonic bath. This was followed by dehydration in ethanol, clearing using xylene, and mounting with cover slips. Imaging of the thin sections was undertaken using a high powered Olympus BX53 microscope, a DP74 camera, and associated Olympus cellSens Life Science Technology software. Images were scanned under a 40 × total magnification (17 mm working distance) and ‘auto-stitched’ using the “Multiple Image Alignment—MIA” tool available from the Olympus cellSens software. Each image was then imported into Adobe Photoshop CC 2014 (replicated recently in Photoshop CC 2020), greyscaled (Black & White), converted to type 16 bit, and thresholded so that Haversian canals throughout cortical bone were ‘enhanced’. This essentially converted cortical bone areas into black pixels and porous bone space into empty/white areas of the image (Fig. 2). Thresholding greyscale images for microscopic quantitative analysis is common practice^122,123^ and can be applied to threshold out Haversian canals^124^. The images were imported into the open access ImageJ vol. 1.52 software for measurements of quantifiable components of the thin sections.

In order to differentiate between porosity producing trabecularisation and Haversian canal density the sections had to be manually segmented. We followed descriptions in^88,89^ where a delimitation between ‘dense’ cortical bone and porosity producing trabecularisation cortical space can be estimated by visually separating the two bone matrices (see red dashed line in Fig. 2). As such, five variables as reported in previous bone porosity studies^123–126^ (some acronyms were modified following the nomenclature standards by Dempster et al^127^) were collected. Total Bone Area (T.B.Ar in mm^2^)^123^ was measured using the ImageJ vol. 1.52 “Polygon” tool by tracing the outer outline endosteal and periosteal borders of the section. Porosity Area (Po.Ar in mm^2^)^124^ was measured using the same tools, but by manually selecting intra-cortical bone regions characterised with abnormal ‘giant pores’ that originated from within the endo-cortical section areas, which we here define as porosity producing trabecularisation. Cortical area (Ct.Ar) was the cortical bone region comprising cortical walls where no porosity producing trabecularisation (Po.Ar) was noted (Ct.Ar = T.B.Ar – Po.Ar). From these measurements, we calculated porosity producing trabecularisation (%Po.Ar = P.Ar/T.B.Ar × 100). Therefore, if no trabecularised regions are consistently observed, this method allows for a null result of Po.Ar and %Po.Ar. From within the Ct.Ar, Haversian canal number (H.N) was counted manually^125,126^ (using the “Multi-Point” tool in ImageJ vol. 1.52 see red dots in Fig. 1). To estimate Haversian canal density (H.Dn)^125,126^, H.N was divided by Ct.Ar to obtain a value per mm^2^. Therefore, we took into consideration two types of bone porosity measures—H.Dn as a proxy for the amount of remodelled bone present in cortical bone, and %Po.Ar which estimates the area of intra-cortical bone affected by trabecularised cortex. Through H.Dn, we worked with an assumption that one Haversian canal represents one secondary osteon. Because cement lines of secondary osteons were not consistently preserved in these archaeological samples, this technique does not account for fragmentary osteons (Fig. 2). The canals that were counted were identified as Haversian canals to the best of our expertise. This excluded micro-features that resembled other pores, which might have occurred as a result of diagenesis. That way, %Po.Ar is exclusively composed of ‘giant’ and coalescing pores, occurring consistently intra-cortically, originating on the endo-cortical part of the sample. These regions should otherwise be filled with dense cortical bone if the individual did not experience significant bone loss. Both measures can provide an insight into a Bone Multi Cellular unit (BMU) activity tunnelling through cortical bone^128^, whereby H.Dn approximates the number of BMUs that once existed per mm^2^, and %Po.Ar indicates prolonged bone resorption through osteoclast-mediated activity.

To provide a qualitative illustration of bone surface topography in relation to the porosity producing trabecularisation, we scanned two contrasting male and female samples (IDs: Sk3.1 and Sk3.2) using an OLS5000 3D laser confocal microscope. Confocal laser scanning microscopy is a recommended technique for characterising porous structures such as bones^129^. The associated OLS5000 2017 LEXT data acquisition and data analysis application software (Olympus LEXT, Japan) (Fig. 1) was then used to apply a heat map of false colours that ranged from red to blue indicating highest to lowest depth, respectively (Fig. 3, S10). This resulted in yellow to green colours indicating low topography (Fig. 3, S11) in the male, and red to blue marking high topography in the female (Fig. 3, S10). We used a 5 × LEXT short working distance objective (20 mm) with a 405 nm violet laser that scans 4,096 pixels along the x-axis, with the zoom as at 1.0x. We used the LEXT automated stitching tool to collectively scan six regions of bone located on the mid-line of the cortical area of the posterior bone quadrant. The scanned area of the female sample was 7192.648 μm long (y-axis) and 4872.699 μm wide (x-axis). The area scanned on the male sample was 7185.926 μm long (y-axis) and 4876.763 μm wide (x-axis). The z-plane depth (height of laser reaching the bone surface) was approximately 3741.925 μm.

Each sample was examined for phosphate and carbonate content using sFTIRM^86,130–136^. Phosphate was selected because it is necessary for bone metabolism, and occurs in skeletal tissue as part of hydroxyapatite crystals^134^. Carbonates (*v*_*2*_ and *v*_*3*_) substitute calcium apatite and influence the properties of crystal in bone, and thus influence bone function at the macroscopic scale^133^. Carbonate in bone is mainly A-type whereby it substitutes for phosphate, but it can also be naturally accompanied by B-type (substituting for hydroxide)^133^. Assessing total (A- and B-type combined) carbonate content, in addition to phosphate, and *v*_2_ carbonate and phosphate ratio of integrated areas under trace, can thus provide an insight into the extent to which calcium phosphate has been resorbed through osteoclast-mediated activity^86,135,136^.

The samples were scanned at the IRM beamline at the Australian Synchrotron facility in Melbourne (Victoria). The scanning technique followed settings reported by Vrahnas et al.^86^ with the exception that we used an attenuated total reflectance (ATR) attachment that allowed a direct contact with bone surface^85,87^. The synchrotron light source provides a highly intense infrared beam that was used to analyse mineral content of bone in situ and the ATR attachment allows the collection of infrared (IR) data from sample sections that are otherwise too thick for conventional IR transmission analysis^87^. The sFTIRM measurement was performed using a Bruker V80v FTIR spectrometer and a Hyperion 3000 IR microscope, which produced high quality spectra in terms of signal-to-noise ratios at 1–2 μm spatial resolution when the synchrotron IR beam coupled to the ATR crystal^87^.

Each sample had four regions of interest (ROIs) identified on the sub-periosteal area of bone, avoiding the endocortical surfaces particularly that they are so extensively affected by abnormal porosity in the Talasiu samples (Fig. 1). Before placing the samples on the microscope stage, the ROIs were identified from images captured at 2 × magnification under a basic dissecting microscope (Fig. 1). We measured carbonates *v*_*2*_ (890–850 cm^−1^) and *v*_*3*_ (1500–1400 cm^−1^), and phosphate (1180–916 cm^−1^), which were then used to calculate carbonate(*v*_*2*_):phosphate ratios (890–850 cm^−1^:1180–916 cm^−1^)^85,86,132,133^. Data analysis was undertaken in OPUS 7.2 and 8.0.19 (Bruker Optik, Germany) by creating integration files and extracting peak height (absorbance/AU) and area under the trace values in each spectrum. Each ROI was scanned for 220 spectra, which totalled 880 spectra per sample, totalling 6820 spectra in the entire sample (this excludes the second region of interest, which was unsuccessfully scanned, therefore deducting 220 spectra in data for individual Sk 3.1).

Once data were inspected, it became apparent that not all spectra were suitable for analysis. This might have occurred as a result of the ATR attachment not making contact with all scanned bone regions. Additionally, as our samples derive from an archaeological context, and are thus impacted by diagenetic processes, diagenetic calcite deposited post-mortem can contribute to carbonate sites of the spectra. To account for these issues, the spectra were inspected and those of poor quality were removed. Our supplemental file (Dataset S1) with raw data reports all spectra collected during the scanning, as well as the datasets following inspection. The inspection involved removing phosphate peak values (and corresponding integrated areas under the peak) of < 0.2. All carbonate spectra values in the negative range to 0.001 were also removed. To further assess for possible diagenesis impacting the carbonate data (sites where calcite can occur), statistical correlations were performed to check how well carbonate *v*_*2*_ and *v*_*3*_ data aligned within each specimen (Table S6, S7). Where no strong correlations were identified, this was taken as an indication that diagenesis might have impacted the data as *v*_*3*_ and *v*_*2*_ values are scattered randomly. We also visually examined the sites of *v*_*3*_ for a strong peak at 1427 cm^−1^ indicating presence of calcite, which should otherwise be replaced by a trough in apatite spectra (creating a doublet)^137^ (Figure S3–S9). Overall, the inspection reduced the number of suitable phosphate spectra to 3344 in males and 844 in females. Carbonate spectra were minimum 1676 (*v*_*2*_) and 2159 (*v*_*3*_) in females, and 3339 (*v*_*2*_) and 3329 (*v*_*3*_) in males (Table 2). The carbonate *v*_*2*_:phosphate ratios calculated from integrated areas under the trace were possible to compute for 846 in females and 2495 in males. We note that all data for Sk12 appeared unsuitable, and so this specimen was excluded from data comparisons.

All data obtained in our study were firstly summarised descriptively. Descriptive comparisons were made within-sample by contrasting results between the sexes, and identifying whether either of the groups showed relatively higher or lower values of data. Next, as our sample size is small (n = 8), we were limited in the choice of statistical analyses to conduct inferential tests. On this basis, we applied non-parametric tests when comparing the sex groups (using a Mann Whitney *U* test^138^) and a Spearman’s *Rho* correlation to test if %Po.Ar was significantly associated with cortical thinning. All inferential statistical testing was conducted in IBM Statistical Package for Social Sciences 26 (SPSS) software. For the sFTIRM spectra inspection through correlations, data normality Kolmogorov–Smirnov tests were performed to check whether non-parametric or parametric correlations tests should be applied (Table S7). This step informed the use of Spearman’s *Rho* correlations as the spectra values were not normally distributed (Table S7, S8). Strong correlations (*Rho* = 0.68–1.00)^139^ were taken as an indication that data can be interpreted to draw conclusions for our research question.

## Supplementary Information

Supplementary Information 1.

Supplementary Video 1.

Supplementary Video 2.

Supplementary Information 2.

## Data Availability

All data generated or analysed during this study are included in this published article (and its Supplementary Information files).
