# Supplementary material for: Bone loss markers in the earliest Pacific Islanders
Source: Sci Rep. 2021 Feb 17;11:3981. doi: 10.1038/s41598-021-83264-3 (PMC7889909; doi:10.1038/s41598-021-83264-3)

**Supplementary Information (SI) for**

BONE LOSS MARKERS IN THE EARLIEST PACIFIC ISLANDERS

Justyna J. Miszkiewicz*, Frédérique Valentin, Christina Vrahnas, Natalie A. Sims, Jitraporn Vongsvivut, Mark J. Tobin, Geoffrey Clark

*E-mail: [Justyna.Miszkiewicz@anu.edu.au](mailto:Justyna.Miszkiewicz@anu.edu.au)

**Table S1 – S8**

**Figure S1-S9**

**Figure S10 (separate GIF file).** Intra-cortical surface scanned using a three-dimensional laser confocal topography scanner Olympus OLS5000 showing extreme porosity in one of the Tongan females (ID: Sk3.1) examined in our study.

**Figure S11 (separate GIF file).** Intra-cortical surface scanned using a three-dimensional laser confocal topography scanner Olympus OLS5000 showing low topography in one of the Tongan males (ID: Sk3.2) examined in our study.

**Dataset S1 (separate file).** All raw data points obtained for each sFTIRM spectrum for phosphate peak height and area, and carbonate *v_2_* and *v_3_.* The file also includes three additional tabs reporting data that were used in our analyses – these are final data used following inspection for poor spectral data.

Table S1. Sex estimates as per Bruzek (2002)^117^, AAD: age-at-death estimates as per Schmitt 2005^118^ (y: years), with brief summaries of possible pathologies observed on the skeletal remains. n/a: not applicable, OA: osteoarthritis.

| **ID** | **Estimated sex (Bruzek, 2002)** | **AAD (Schmitt, 2005)** | **Health status** |
| --- | --- | --- | --- |
| BG2 | Female | Adult (>50y) | Degenerative lesions at both acetabula, OA in several joints |
| Sk3.1 | Female | Adult (>60y) | Antemortem tooth loss on lower dentition, OA in several joints, degenerative lesions on vertebral column |
| Sk3.2 | Male | Adult  (>30y) | OA in several joints, traumatic hematoma (?) |
| Sk5 | Male | Adult  (20-39y) | Degenerative lesions at both acetabula, OA in several joints |
| Sk9.2 | Probable female | Adult  (auricular surface damage) | OA in bones of extremities, degenerative lesions on vertebral column, cystic lesions periarticular at left first metatarsal joint |
| Sk9.3 | Probable male | Adult  (no auricular surface) | Antemortem tooth loss, beginning of ankylosis at right sacro iliac joint, degenerative lesions at vertebral column, OA in several joints, traumatic hematoma |
| Sk12 | Probable female | Adult (>50y) | n/a |
| Sk14 | Male | Adult (>50y) | Beginning of ankylosis at sacro iliac joint, degenerative lesions at vertebral column, OA in several joints, traumatic hematoma |

Table S2. Raw data for the entire sample. Sex estimates as per Bruzek (2002)^117^ (the ‘probable’ classifications hereafter referred to as either male or female; refer to Table S1 for corresponding age-at-death). A-P.dm: antero-posterior midshaft diameter in mm, M-L.dm: medio-lateral midshaft diameter in mm, M.Circ: midshaft circumference in mm, Ct.Ar: cortical area in mm^2^, H.N: Haversian canal number, H.Dn: Haversian canal density per mm^2^ (H.N/Ct/Ar), Po.Ar: abnormal porosity in mm^2^, T.B.Ar: total bone area in mm^2^, %Po.Ar: percent abnormal porosity of thin section (Po.Ar/T.B.Ar x 100).

| **ID** | **Bone** | **Estimated sex** | **A-P.dm** | **M-L.dm** | **M.Circ** | **Ct.Ar** | **H.N** | **H.Dn** | **Po.Ar** | **T.B.Ar** | **%Po.Ar** |
| --- | --- | --- | --- | --- | --- | --- | --- | --- | --- | --- | --- |
| BG2 | Right femur | Female | 25.28 | 23.35 | 76.00 | 49.948 | 1110 | 22.223 | 23.386 | 73.334 | 31.890 |
| Sk3.1 | Right femur | Female | 28.93 | 24.87 | 86.00 | 62.088 | 817 | 13.159 | 62.667 | 124.755 | 50.232 |
| Sk3.2 | Right femur | Male | 33.87 | 29.27 | 101.00 | 155.951 | 2437 | 15.627 | 12.210 | 168.161 | 7.261 |
| Sk5 | Right femur | Male | 27.72 | 22.17 | 86.00 | 62.603 | 1302 | 20.798 | 0.000 | 62.603 | 0.000 |
| Sk9.2 | Left femur | Probable female | 29.30 | 26.54 | 81.00 | 42.592 | 631 | 14.815 | 36.284 | 78.876 | 46.001 |
| Sk9.3 | Right femur | Probable male | 29.00 | 27.34 | 87.00 | 39.263 | 649 | 16.530 | 42.184 | 81.447 | 51.793 |
| Sk12 | Right femur | Probable female | 25.54 | 23.26 | 76.00 | 51.328 | 627 | 12.216 | 8.292 | 59.620 | 13.908 |
| Sk14 | Right femur | Male | 32.41 | 26.92 | 95.00 | 102.582 | 1726 | 16.826 | 4.494 | 107.076 | 4.197 |

**Table S3.** Descriptive data per each individual reporting sFTIRM phosphate in absorbance (Au) units. Sk12 is excluded due to phosphate peak data not meeting our criteria (> 0.2) for inclusion in analyses likely due to impact of diagenesis – see Methods.

| **Estimated sex** | **ID** | **Variable** | **N** | **Min.** | **Max.** | **Mean** | **SD** |
| --- | --- | --- | --- | --- | --- | --- | --- |
| **Female** | BG2 | Phosphate peak (Au) | 356 | 0.201 | 1.004 | 0.446 | 0.168 |
|  |  | Phosphate area (Au) | 356 | 15.630 | 59.790 | 31.978 | 10.281 |
|  | Sk3.1 | Phosphate peak (Au) | 251 | 0.203 | 1.139 | 0.600 | 0.189 |
|  |  | Phosphate area (Au) | 251 | 15.087 | 71.381 | 41.300 | 11.630 |
|  | Sk9.2 | Phosphate peak (Au) | 241 | 0.202 | 0.817 | 0.410 | 0.159 |
|  |  | Phosphate area (Au) | 241 | 15.465 | 58.586 | 31.205 | 10.721 |
| **Male** | Sk14 | Phosphate peak (Au) | 668 | 0.201 | 1.011 | 0.485 | 0.161 |
|  |  | Phosphate area (Au) | 668 | 15.045 | 63.060 | 34.284 | 9.961 |
|  | Sk3.2 | Phosphate peak (Au) | 429 | 0.201 | 1.156 | 0.513 | 0.207 |
|  |  | Phosphate area (Au) | 429 | 14.937 | 72.184 | 35.559 | 12.297 |
|  | Sk5 | Phosphate peak (Au) | 794 | 0.207 | 0.918 | 0.538 | 0.151 |
|  |  | Phosphate area (Au) | 794 | 16.148 | 60.173 | 37.147 | 8.913 |
|  | Sk9.3 | Phosphate peak (Au) | 605 | 0.200 | 0.922 | 0.435 | 0.160 |
|  |  | Phosphate area (Au) | 605 | 15.295 | 63.358 | 32.158 | 10.305 |

**Table S4**. Descriptive data reporting sFTIRM carbonate *v*_2_ data in absorbance (Au) units per individual and sex. Sk12 is excluded due to carbonate data likely being impacted by diagenesis.

| **Estimated sex** | **ID** | **Variable** | **N** | **Min.** | **Max.** | **Mean** | **SD** |
| --- | --- | --- | --- | --- | --- | --- | --- |
| **Female** | BG2 | *v*_2_ height (Au) | 628 | 0.001 | 0.064 | 0.023 | 0.015 |
|  |  | *v*_2_ area (Au) | 628 | 0.001 | 1.295 | 0.398 | 0.282 |
|  | Sk3.1 | *v*_2_ height (Au) | 357 | 0.001 | 0.216 | 0.033 | 0.024 |
|  |  | *v*_2_ area (Au) | 357 | 0.002 | 2.899 | 0.568 | 0.401 |
|  | Sk9.2 | *v*_2_ height (Au) | 691 | 0.001 | 0.079 | 0.018 | 0.015 |
|  |  | *v*_2_ area (Au) | 691 | 0.002 | 1.504 | 0.297 | 0.281 |
| **Male** | Sk14 | *v*_2_ height (Au) | 834 | 0.001 | 0.087 | 0.036 | 0.017 |
|  |  | *v*_2_ area (Au) | 834 | 0.003 | 1.445 | 0.625 | 0.301 |
|  | Sk3.2 | *v*_2_ height (Au) | 786 | 0.001 | 0.104 | 0.026 | 0.019 |
|  |  | *v*_2_ area (Au) | 786 | 0.005 | 1.361 | 0.454 | 0.332 |
|  | Sk5 | *v*_2_ height (Au) | 864 | 0.001 | 0.078 | 0.037 | 0.013 |
|  |  | *v*_2_ area (Au) | 864 | 0.006 | 1.461 | 0.644 | 0.228 |
|  | Sk9.3 | *v*_2_ height (Au) | 855 | 0.002 | 0.089 | 0.033 | 0.017 |
|  |  | *v*_2_ area (Au) | 855 | 0.011 | 1.625 | 0.571 | 0.307 |

Table S5. Descriptive data reporting sFTIRM carbonate *v*_3_ data in absorbance (Au) units per individual and sex. Sk12 is excluded due to carbonate data likely being impacted by diagenesis.

| **Estimated sex** | **ID** | **Variable** | **N** | **Min.** | **Max.** | **Mean** | **SD** |
| --- | --- | --- | --- | --- | --- | --- | --- |
| **Female** | BG2 | *v*_3_ height (Au) | 830 | 0.001 | 0.033 | 0.010 | 0.007 |
|  |  | *v*_3_ area (Au) | 820 | 0.007 | 2.014 | 0.485 | 0.432 |
|  | Sk3.1 | *v*_3_ height (Au) | 578 | 0.001 | 0.050 | 0.013 | 0.010 |
|  |  | *v*_3_ area (Au) | 557 | 0.004 | 2.873 | 0.596 | 0.631 |
|  | Sk9.2 | *v*_3_ height (Au) | 751 | 0.001 | 0.041 | 0.010 | 0.007 |
|  |  | *v*_3_ area (Au) | 694 | 0.001 | 2.294 | 0.367 | 0.419 |
| **Male** | Sk14 | *v*_3_ height (Au) | 843 | 0.002 | 0.042 | 0.017 | 0.008 |
|  |  | *v*_3_ area (Au) | 828 | 0.001 | 2.435 | 0.890 | 0.497 |
|  | Sk3.2 | *v*_3_ height (Au) | 777 | 0.001 | 0.352 | 0.014 | 0.017 |
|  |  | *v*_3_ area (Au) | 741 | 0.003 | 8.510 | 0.666 | 0.620 |
|  | Sk5 | *v*_3_ height (Au) | 865 | 0.002 | 0.041 | 0.021 | 0.007 |
|  |  | *v*_3_ area (Au) | 862 | 0.013 | 2.032 | 1.085 | 0.419 |
|  | Sk9.3 | *v*_3_ height (Au) | 844 | 0.001 | 0.043 | 0.015 | 0.008 |
|  |  | *v*_3_ area (Au) | 833 | 0.002 | 2.190 | 0.731 | 0.464 |

**Table S6.** Descriptive data reporting sFTIRM carbonate *v_2_*:phosphate ratio data (unitless) per individual and sex. Sk12 is excluded due to data likely being impacted by diagenesis.

| **Estimated sex** | **ID** | **Variable** | **N** | **Min.** | **Max.** | **Mean** | **SD** |
| --- | --- | --- | --- | --- | --- | --- | --- |
| **Female** | BG2 | RATIO | 356 | 0.004 | 0.030 | 0.018 | 0.004 |
|  | Sk3.1 | RATIO | 249 | 0.003 | 0.143 | 0.019 | 0.010 |
|  | Sk9.2 | RATIO | 241 | 0.001 | 0.055 | 0.019 | 0.005 |
| **Male** | Sk14 | RATIO | 667 | 0.004 | 0.038 | 0.021 | 0.003 |
|  | Sk3.2 | RATIO | 429 | 0.001 | 0.037 | 0.019 | 0.004 |
|  | Sk5 | RATIO | 794 | 0.009 | 0.031 | 0.019 | 0.002 |
|  | Sk9.3 | RATIO | 605 | 0.008 | 0.033 | 0.022 | 0.003 |

Table S7. Data distribution tests in the carbonate *v_2_* and *v_3_* peaks in absorbance units (Au) to inform subsequent correlations. The null hypothesis was that the data were normally distributed (*p* > 0.05), but neither of the variables were normally distributed (*p* < 0.05). The subsequent correlations (Table S7) were performed using non-parametric Spearman’s *Rho* tests.

| Variables and sample | K–S | df | *p* |
| --- | --- | --- | --- |
| Carbonate *v_2_* peak (Au) - whole sample | 0.081 | 5332 | <0.0001 |
| Carbonate *v_2_* peak (Au) – BG2 | 0.084 | 628 | <0.0001 |
| Carbonate *v_2_* peak (Au) – Sk12 | 0.086 | 317 | <0.0001 |
| Carbonate *v_2_* peak (Au) – Sk14 | 0.051 | 834 | <0.0001 |
| Carbonate *v_2_* peak (Au) – Sk3.1 | 0.090 | 357 | <0.0001 |
| Carbonate *v_2_* peak (Au) – Sk3.2 | 0.122 | 786 | <0.0001 |
| Carbonate *v_2_* peak (Au) – Sk5 | 0.071 | 864 | <0.0001 |
| Carbonate *v_2_* peak (Au) – Sk9.2 | 0.158 | 691 | <0.0001 |
| Carbonate *v_2_* peak (Au) – Sk9.3 | 0.038 | 855 | 0.005 |
| Carbonate *v_3_* peak (Au) – whole sample | 0.121 | 6552 | <0.0001 |
| Carbonate *v_3_* peak (Au) – BG2 | 0.131 | 830 | <0.0001 |
| Carbonate *v_3_* peak (Au) – Sk12 | 0.039 | 764 | 0.007 |
| Carbonate *v_3_* peak (Au) – Sk14 | 0.049 | 843 | <0.0001 |
| Carbonate *v_3_* peak (Au) – Sk3.1 | 0.232 | 578 | <0.0001 |
| Carbonate *v_3_* peak (Au) – Sk3.2 | 0.221 | 777 | <0.0001 |
| Carbonate *v_3_* peak (Au) – Sk5 | 0.062 | 865 | <0.0001 |
| Carbonate *v_3_* peak (Au) – Sk9.2 | 0.205 | 751 | <0.0001 |
| Carbonate *v_3_* peak (Au) – Sk9.3 | 0.070 | 844 | <0.0001 |

Table S8. Results from Spearman’s *Rho* tests for correlations between *v_2_* and *v_3_* data to check whether the values correlated. Where they do not, we assume the spectral data had been altered by diagenesis. These results inform which *v_2_* and *v_3_* we subsequently reported.

| Correlated variables | *Rho* | *p* | Decision |
| --- | --- | --- | --- |
| Carbonate *v_2_* and *v_3_* peak (Au) – whole sample | 0.917 | <0.0001 | Interpret & check per skeleton |
| Carbonate *v_2_* and *v_3_* peak (Au), BG2 | 0.883 | <0.0001 | Interpret |
| Carbonate *v_2_* and *v_3_* peak (Au), Sk12 | -0.50 | 0.402 | Exclude due to possible impact of diagenesis |
| Carbonate *v_2_* and *v_3_* peak (Au), Sk14 | 0.910 | <0.0001 | Interpret |
| Carbonate *v_2_* and *v_3_* peak (Au), Sk3.1 | 0.942 | <0.0001 | Interpret |
| Carbonate *v_2_* and *v_3_* peak (Au), Sk3.2 | 0.875 | <0.0001 | Interpret |
| Carbonate *v_2_* and *v_3_* peak (Au), Sk5 | 0.836 | <0.0001 | Interpret |
| Carbonate *v_2_* and *v_3_* peak (Au), Sk9.2 | 0.837 | <0.0001 | Interpret |
| Carbonate *v_2_* and *v_3_* peak (Au), Sk9.3 | 0.923 | <0.0001 | Interpret |

**Figure S1.** Posterior femur sample from individual Sk9.3 estimated as a probable male, showing extensive intra-cortical porosity. The femur midshaft cross-section is schematic and the images are not-to-scale.

**
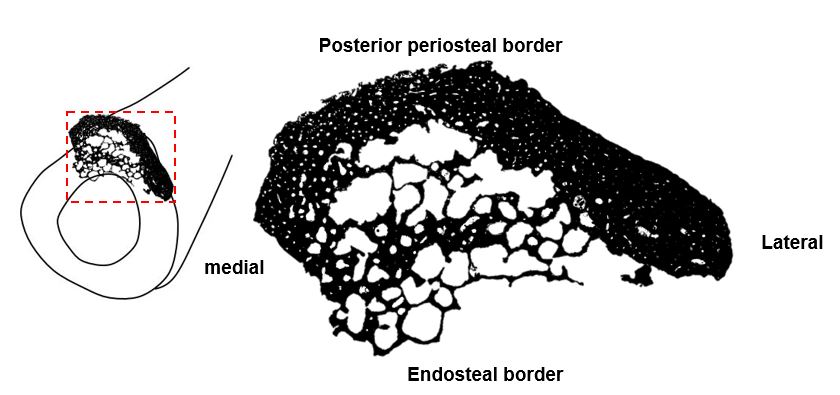
**

**Figure S2.** Mandible of estimated female (ID: Sk3.1) showing closed up and remodelled tooth sockets possibly as a result of ante-mortem tooth loss due to old age. This individual was assigned an age-at-death category of 50+ years old.


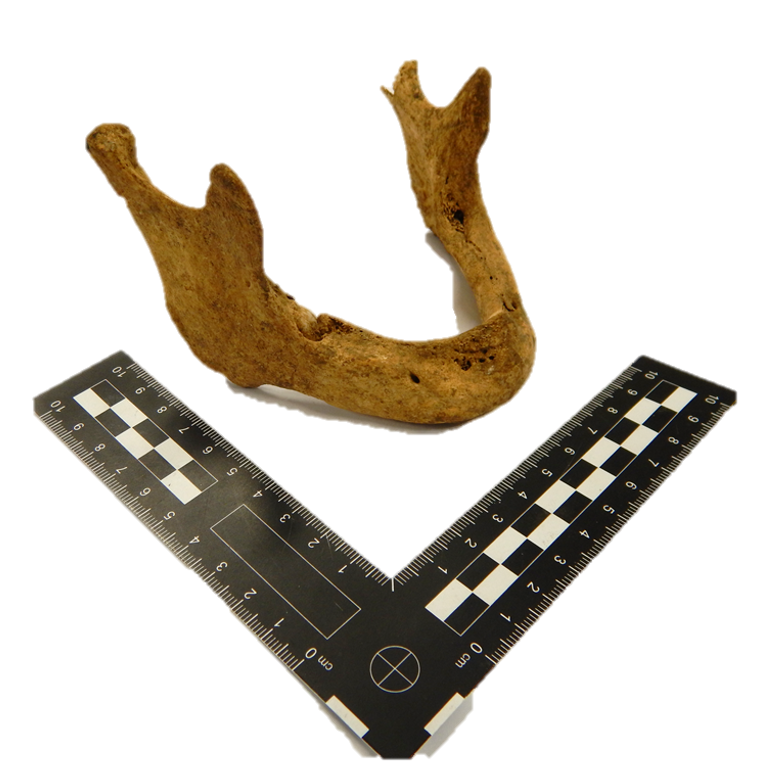


**Figure S3.** Sample spectra with integrated sites (zoomed in on the right) for carbonate *v_3_* in Sk3.2 (region of interest, ROI = 1).


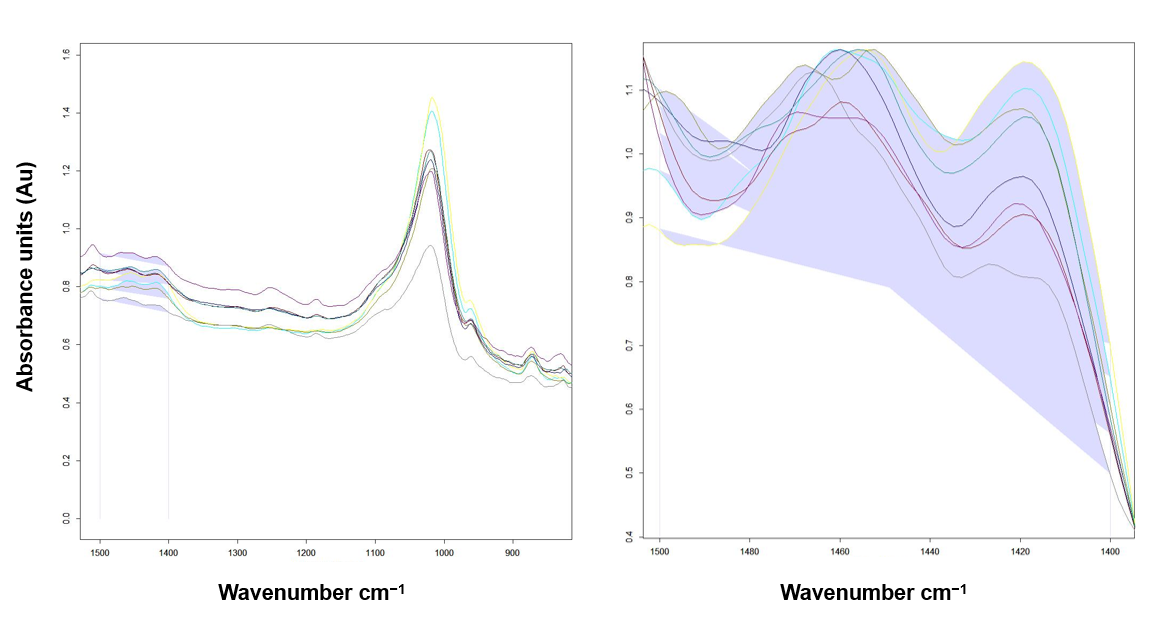


**Figure S4.** Sample spectra with integrated sites (zoomed in on the right) for carbonate *v_3_* in Sk3.1 (region of interest, ROI = 1).


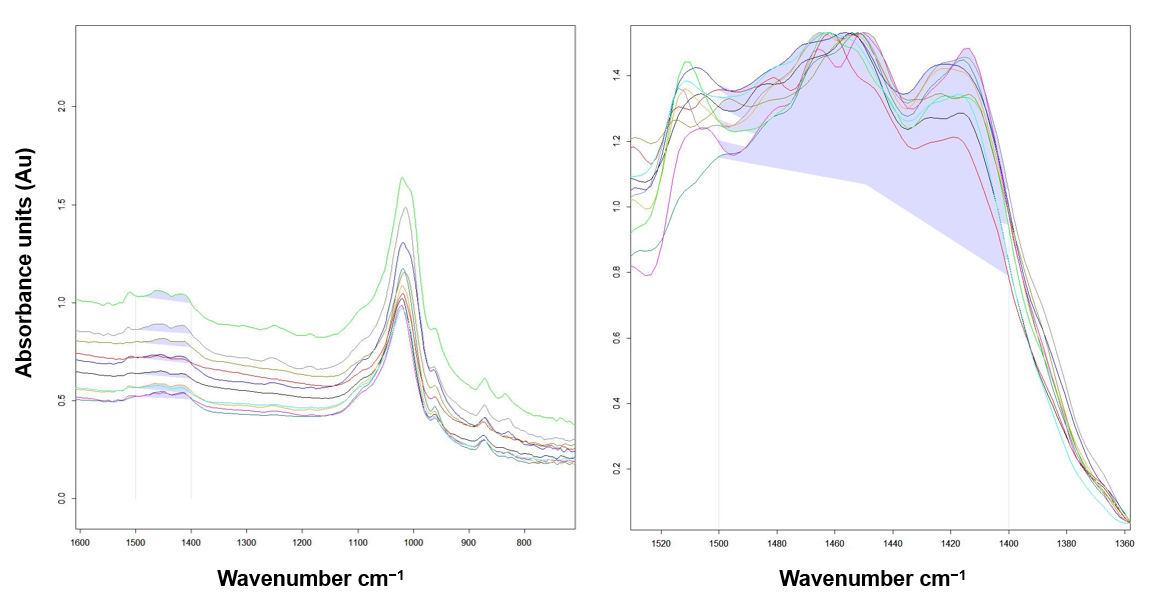


**Figure S5.** Sample spectra with integrated sites (zoomed in on the right) for carbonate *v_3_* in Sk9.3 (region of interest, ROI = 1).


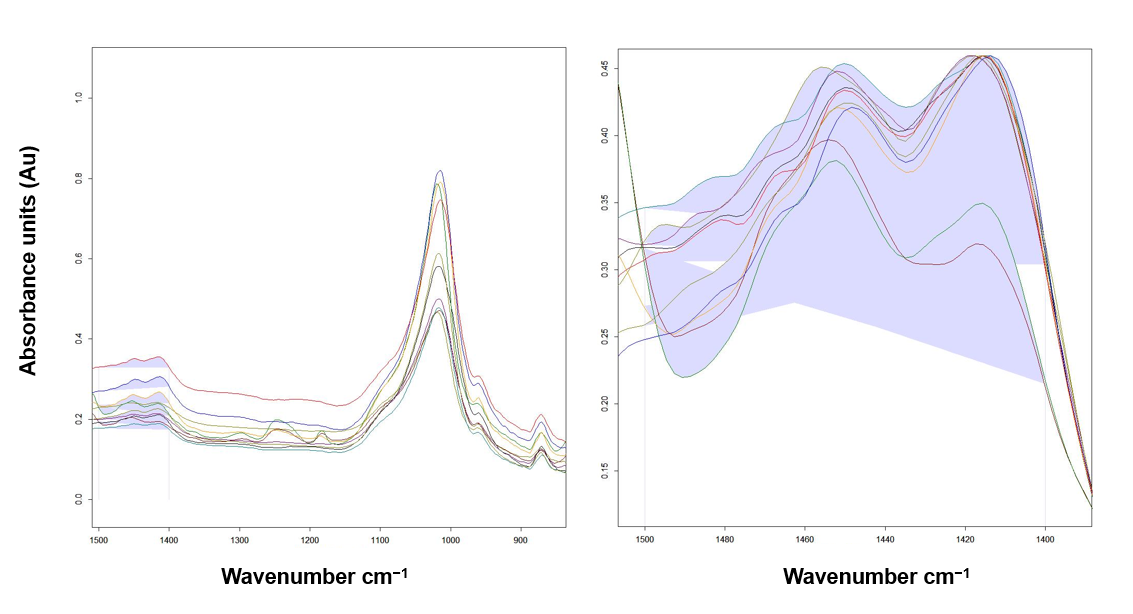


**Figure S6.** Sample spectra with integrated sites (zoomed in on the right) for carbonate *v_3_* in Sk14 (region of interest, ROI = 1).


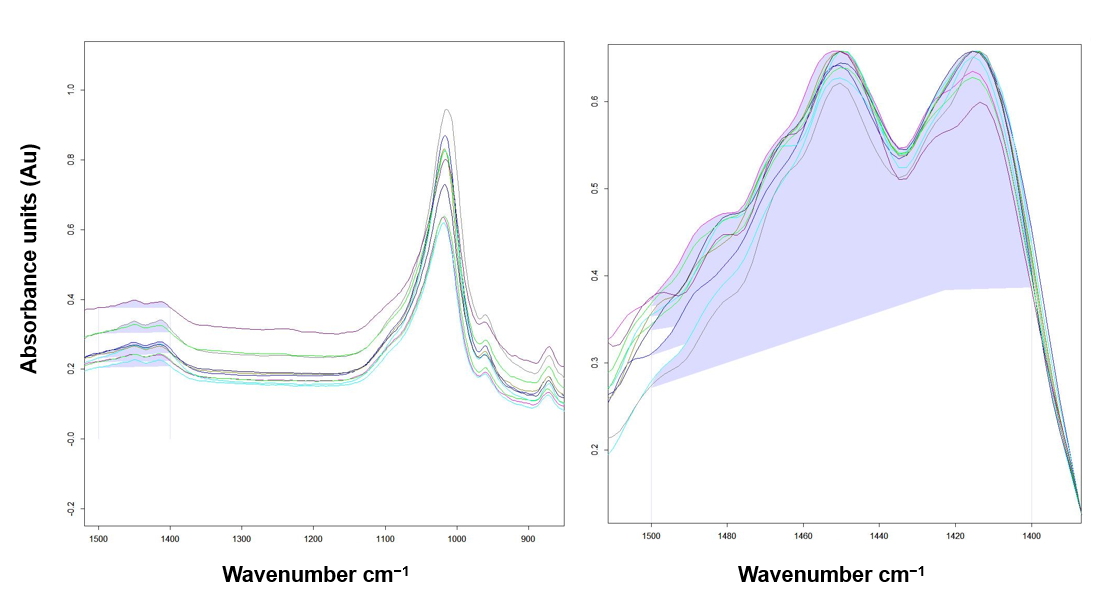


**Figure S7.** Sample spectra with integrated sites (zoomed in on the right) for carbonate *v_3_* in BG2 (region of interest, ROI = 4).


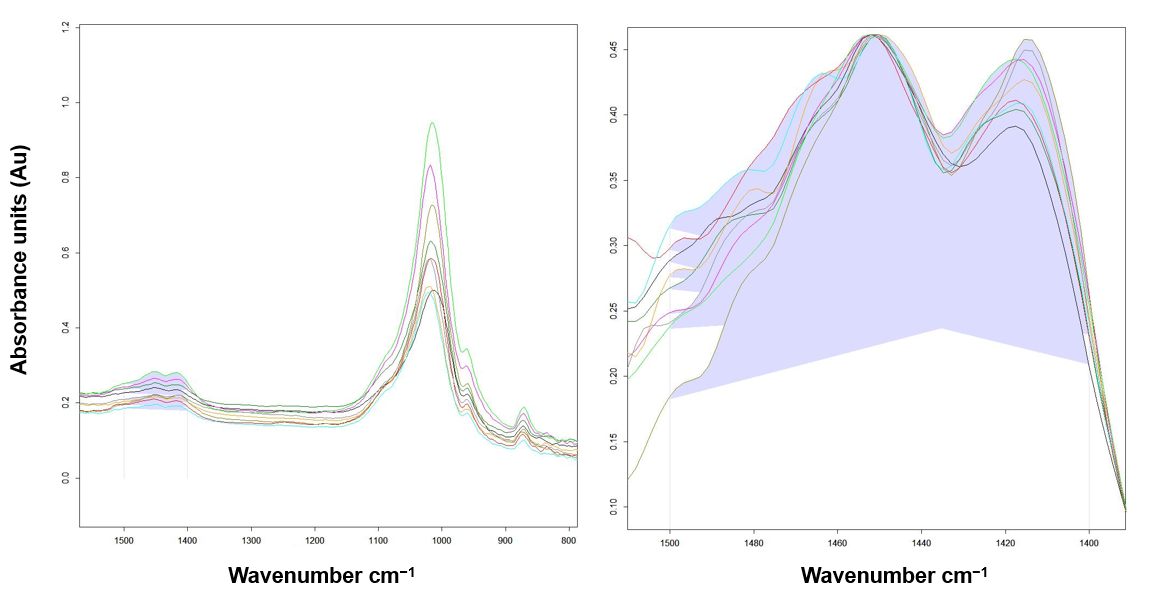


**Figure S8.** Sample spectra with integrated sites (zoomed in on the right) for carbonate *v_3_* in Sk5 (region of interest, ROI = 2).


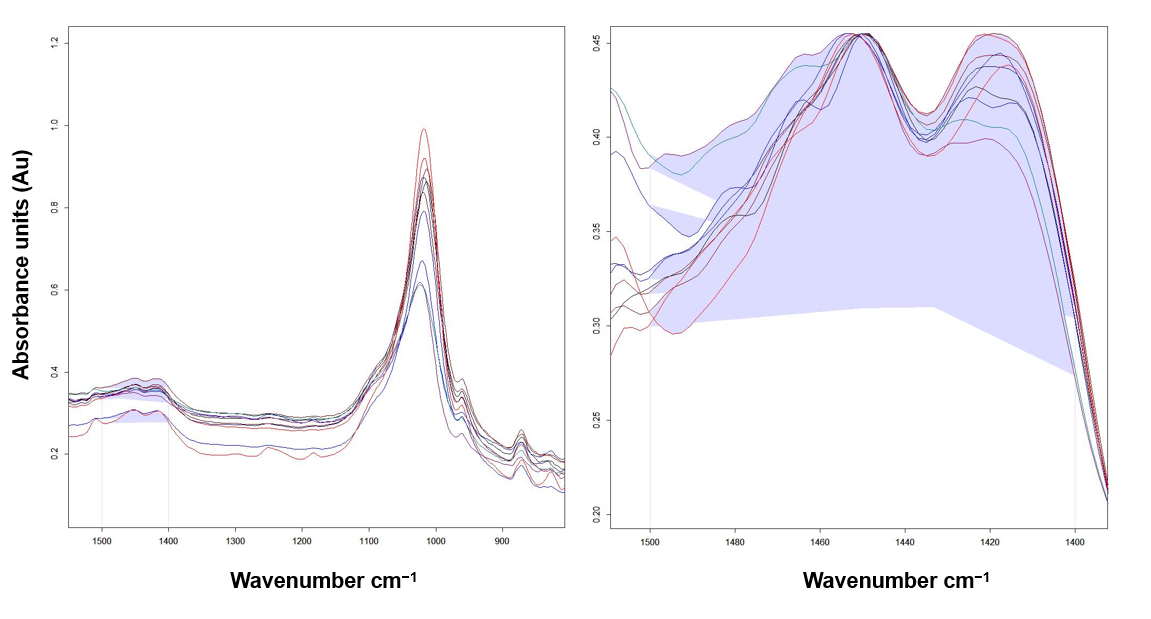


**Figure S9.** Sample spectra with integrated sites (zoomed in on the right) for carbonate *v_3_* in Sk9.2 (region of interest, ROI = 4).


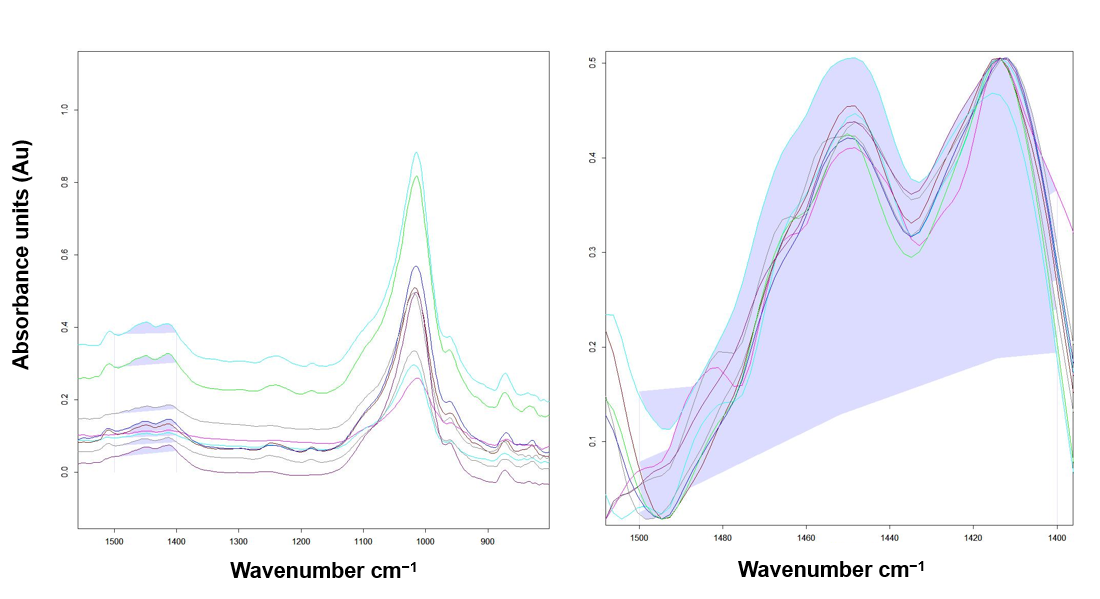

Supplement: Supplementary file 1 — Supplementary Information 1. [file 41598_2021_83264_MOESM1_ESM.docx]
